# Emergency cardiac imaging for coronavirus disease 2019 (COVID-19) in practice: a case of takotsubo stress cardiomyopathy

**DOI:** 10.1186/s12947-021-00251-4

**Published:** 2021-08-24

**Authors:** Oriana Belli, Maddalena Ardissino, Maurizio Bottiroli, Francesco Soriano, Calogero Blanda, Jacopo Oreglia, Michele Mondino, Antonella Moreo

**Affiliations:** 1 Division, of Cardiology, ‘A. De Gasperis’ Cardio Center, ASST Grande Ospedale Metropolitano Niguarda, Piazza Ospedale Maggiore 3, 20162 Milan, Italy; 2 Department of Medicine, Imperial College London, Exhibition Road, London, UK; 3 Division of Anaesthetics and Intensive Care, ASST Grande Ospedale Metropolitano Niguarda, Milan, Italy

**Keywords:** Tako-tsubo, Coronavirus, COVID-19, Cardiomyopathy, Stress

## Abstract

**Background:**

Cardiovascular complications of severe acute respiratory distress syndrome coronavirus 2 (SARS-CoV2) are known to be associated with poor outcome. A small number of case series and reports have described cases of myocarditis and ischaemic events, however, knowledge on the aetiology of acute cardiac failure in SARS-CoV2 remains limited. We describe the occurrence and risk stratification imaging correlates of ‘takotsubo’ stress cardiomyopathy presenting in a patient with Coronavirus Disease 2019 (COVID-19) in the intensive care unit.

**Case summary:**

An intubated 53-year old patient with COVID19 suffered acute haemodynamic collapse in the intensive care unit, and was thus investigated with transthoracic echocardiography (TTE), 12-lead electrocardiograms (ECG) and serial troponins and blood tests, and eventually coronary angiography due to clinical suspicion of ischaemic aetiology. Echocardiography revealed a reduced ejection fraction, with evident extensive apical akinesia spanning multiple coronary territories. Troponins and NT-proBNP were elevated, and ECG revealed ST elevation: coronary angiography was thus performed. This revealed no significant coronary stenosis. Repeat echocardiography performed within the following week revealed a substantial recovery of ejection fraction and wall motion abnormalities. Despite requirement of a prolonged ICU stay, the patient now remains clinically stable, and is on spontaneous breathing.

**Conclusion:**

This case report presents a case of takotsubo stress cardiomyopathy occurring in a critically unwell patient with COVID19 in the intensive care setting. Stress cardiomyopathy may be an acute cardiovascular complication of COVID-19 infection. In the COVID19 critical care setting, urgent bedside echocardiography is an important tool for initial clinical assessment of patients suffering haemodynamic compromise.

**Supplementary Information:**

The online version contains supplementary material available at 10.1186/s12947-021-00251-4.

## Learning points

Tako-tsubo stress cardiomyopathy can be an acute cardiovascular complication of COVID-19 infection; in the COVID19 critical care setting, urgent bedside echocardiography is an important tool for initial clinical assessment of patients suffering haemodynamic compromise.

## Introduction

The novel Severe Acute Respiratory Distress Syndrome coronavirus 2 (SARS-CoV2) has caused a huge mortality and morbidity burden worldwide. The clinical course of the coronavirus 2019 (COVID19) disease is known to mostly follow a progressive respiratory failure. Knowledge on the patterns and characteristics of cardiac involvement in COVID19 remains sparse but is rapidly growing [1, 2].

## Case presentation

**Fig. 1:**
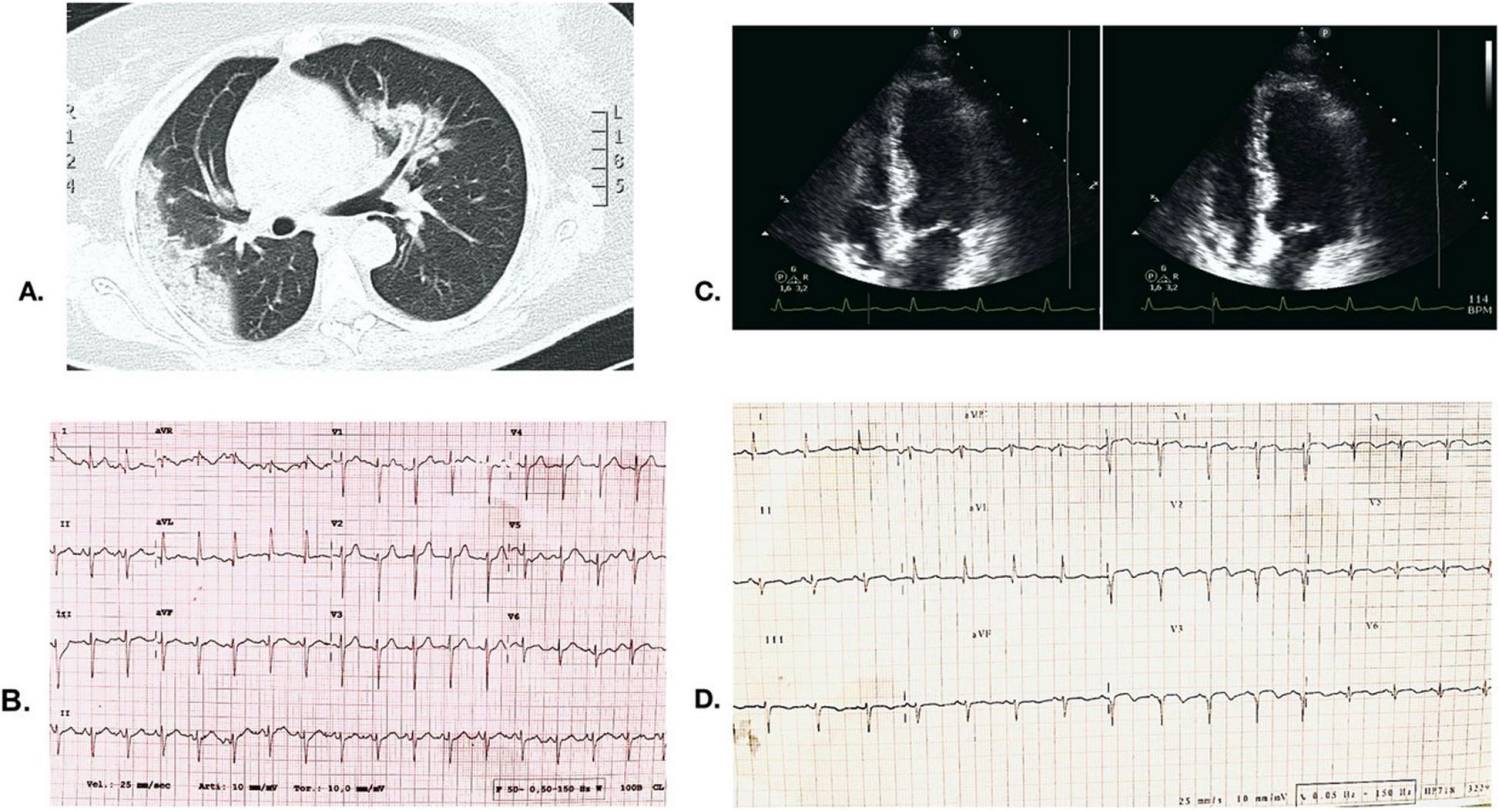
Initial imaging and clinical investigation results. **a** Right sided ground glass opacities and left sided dense ground glass with consolidation on CT chest, consistent with COVID19. **b** Admission ECG displayed no acute ST segment of T wave changes, and a normal QTc. **c** Extensive apical akinesia and ballooning involving medial anterior, lateral, inferior and septal segments. LV systolic function was globally impaired (LVEF 30%). **d** ECG indicating ST elevation with biphasic T waves and Q waves most prominent in the anterolateral segment (leads V_1-6_)

**Fig. 2:**
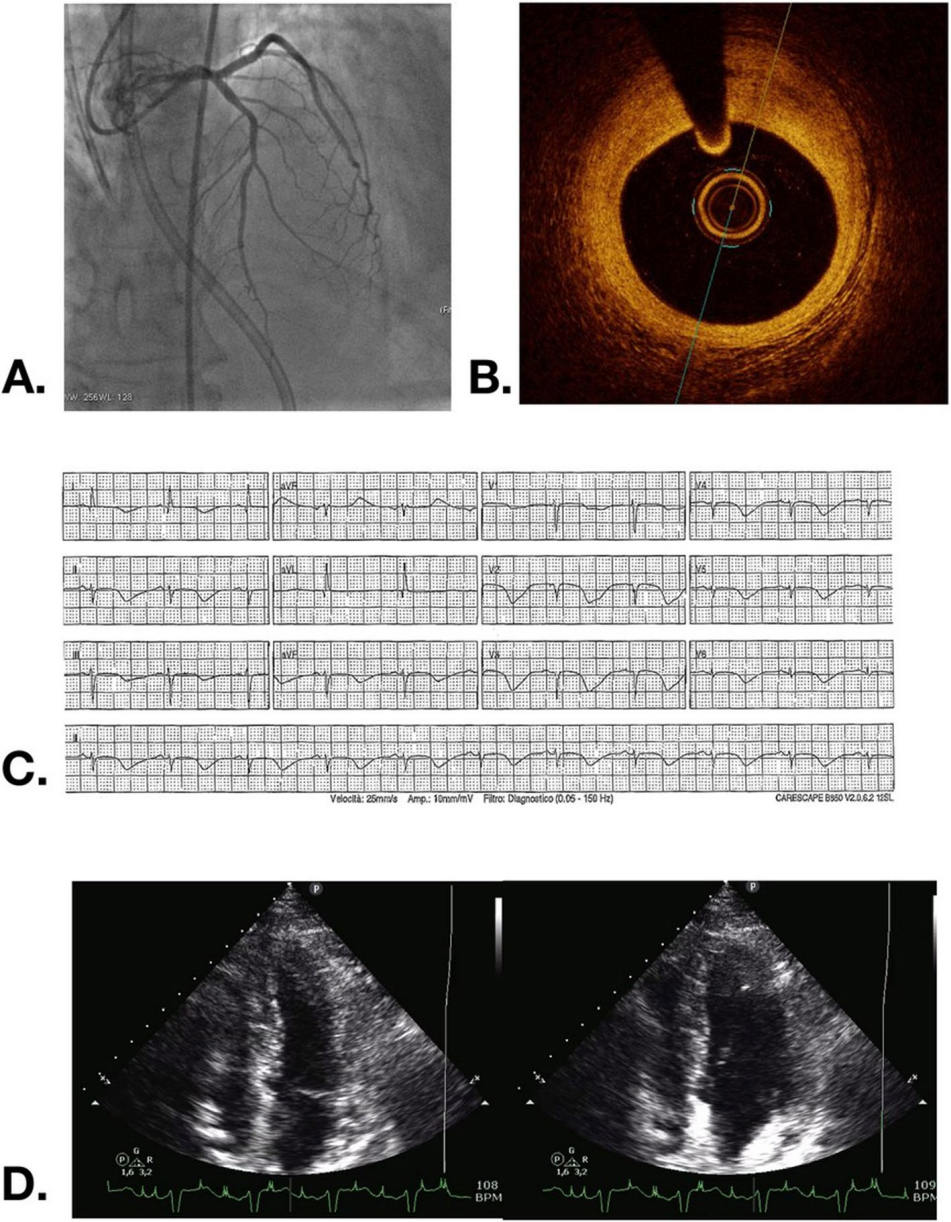
Cardiovascular imaging and angiography results. **a** Coronary angiography showed no obstructive coronary artery disease with normal flow (TIMI 3) and mild atherosclerotic plaque in the middle tract of LAD. **b** Optical coherence tomography (OCT) of LAD confirmed presence of fibro-lipidic plaque in the middle tract of LAD with positive remodeling without signs of erosion, ulceration or dissection. **c** Repeat ECG showed characteristic prolonged QT interval (> 600 ms) and typical deep T wave inversion. **d** Improvement in wall motion on repeat echocardiogram

A 53 year old female with a background of chronic kidney disease Stage III was admitted with typical symptoms for COVID19 and consistent changes on chest CT (Fig. 1a). Admission ECG was unremarkable (Fig. 1b). She was confirmed to be SARS-CoV2 positive and was started on hydroxychloroquine, as this constituted part of the management protocol for COVID-19 in the early stages of the pandemic. After a short trial of CPAP, she required intubation and was moved to the intensive care unit (ICU). On the second day in the ICU, she became hemodynamically unstable. Troponin T was 236 ng/L to 189 ng/L, and NT-proBNP was elevated at 18,732 ng/L. Urgent TTE identified complete apical ballooning and extensive akinesia spanning multiple coronary territories with a global LV systolic function impairment (LVEF 30%) (Fig. 1c; Video 1). ECG revealed ST elevation with biphasic T waves and Q waves (Fig. 1d); urgent angiography was thus performed. Angiography identified a non-significant 30% stenosis of the left anterior descending coronary artery with otherwise smooth coronary arteries (Fig. 2a). On OCT, the plaque was long and fibrinous, with no evidence of instability or erosions (Fig. 2b). In the following days, TnT settled to 36–40 ng/L and NT-proBNP reduced to 3,628 ng/L. A diagnosis of takotsubo stress cardiomyopathy was made in line with Mayo criteria and upon further investigations: serial ECG showed development of characteristic prolonged QT interval (> 600 ms) and typical deep T wave inversion (Fig. 2c). Hydroxychloroquine was immediately stopped; as it was supposed that it could have contributed to the prolongation of the QT interval. Repeat TTE the following week while still intubated revealed marked improvement of left ventricular systolic function and motion abnormalities (Fig. 2d; Video 2). A summary of the timeline of events is provided in Supplementary Figure 1.

## Discussion

Knowledge on the patterns and characteristics of cardiac involvement in COVID19 remains sparse but is rapidly growing [1–4]. We describe a case of stress cardiomyopathy in a COVID19 patient in the ICU setting; and highlight the critical role of timely and targeted cardiac imaging for its diagnosis and management. Takotsubo stress cardiomyopathy is a well-recognized clinical entity characterised by sudden onset, non-regional left ventricular dysfunction associated with typical ECG patterns after recent physical or emotional stress. It generally carries a good clinical prognosis with high rates of relatively fast clinical and echocardiographic recovery [5].

This case sparks a number of important discussion points. Firstly, the significance of the otherwise rare takotsusbo stress cardiomyopathy in the current COVID19 pandemic is unknown. Stress cardiomyopathy is known to be a possible but though rare cause of haemodynamic instability and acute heart failure, but its relative prevalence among COVID19 patients, and thus its clinical significance within the current pandemic, are yet unknown. Already an elusive diagnosis in usual times, it risks being further underdiagnosed (and thus undertreated) in the current climate of protective equipment and staffing shortages. Currently, we know that it is more common in patients with socioeconomic stressors [1], and that it mainly occurs in females, in the first 3–4 days of hospitalization, and that among reported cases it carries a rate of recovery of 90% [2].

This case thus highlights the crucial role of urgent echocardiography; as it played a key role in dictating further investigations and management for the patient. In line with recommendations from the EACVI [6], urgent investigations including bedside echocardiography are of paramount importance and priority in patients in whom it will impact clinical management decisions. In this case, the finding of impaired systolic function with akinesia in the apical segment led to urgent further evaluation with coronary angiography to exclude an acute coronary event, and upon finding of unobstructed coronary arteries, the patient was continued on conservative vasopressors for haemodynamic support with view to wean off as tolerated, and subsequent introduction of bisoprolol. It is important to also note that in this particular case, the differential diagnosis of myocarditis remains an important alternative which would have required endomyocardial biopsy to be ruled out. The patient ultimately made a good clinical recovery, but required prolonged intensive care stay, ionotropic support and ventilation. Despite the known association between cardiovascular involvement and poor outcomes of COVID19, there is still a paucity of data characterising the cardiovascular complications of the disease. We highlight the importance further research and reporting of clinical cases in order to improve their early detection and enable timely, appropriate management of COVID19-related cardiovascular complications.

## Supplementary Information

**Additional file 1: Video 1**. Left ventricle (LV) dysfunction (LVEF 30%) with apical akinesia and ballooning, with preserved motion of the basal segments.

**Additional file 2: Video 2**. Resolution of motion abnormalities and improvement in ejection fraction on repeat echocardiogram.

**Additional file 3: Supplementary Figure 1**. Timeline of clinical progression and events.

## Data Availability

All data generated and analyzed in this study is available from the corresponding author on reasonable request.
