# Supplementary figures and images for: Emergency cardiac imaging for coronavirus disease 2019 (COVID-19) in practice: a case of takotsubo stress cardiomyopathy
Source: Cardiovasc Ultrasound. 2021 Aug 24;19:31. doi: 10.1186/s12947-021-00251-4 (PMC8383239; doi:10.1186/s12947-021-00251-4)

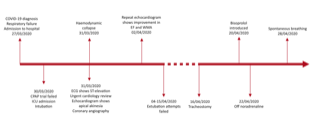

Supplement: Supplementary file 3 — Additional file 3: Supplementary Figure 1. Timeline of clinical progression and events. [file 12947_2021_251_MOESM3_ESM.tiff]
